# Toward a Procedure for the Template Free Growth of Te Nanowires Across an Insulator by Electrodeposition

**DOI:** 10.1021/acs.jpcc.4c05915

**Published:** 2024-10-22

**Authors:** Alexander W. Black, Nema M. Abdelazim, Yasir J. Noori, Yisong Han, Nikolay Zhelev, Shibin Thomas, Wenjian Zhang, Gillian Reid, Richard Beanland, C. H. Kees de Groot, Philip N. Bartlett

**Affiliations:** † School of Chemistry, University of Southampton, Southampton SO17 1BJ, U.K.; ‡ School of Electronics and Computer Science, University of Southampton, Southampton SO17 1BJ, U.K.; § Department of Physics, University of Warwick, Coventry CV4 7AL, U.K.

## Abstract

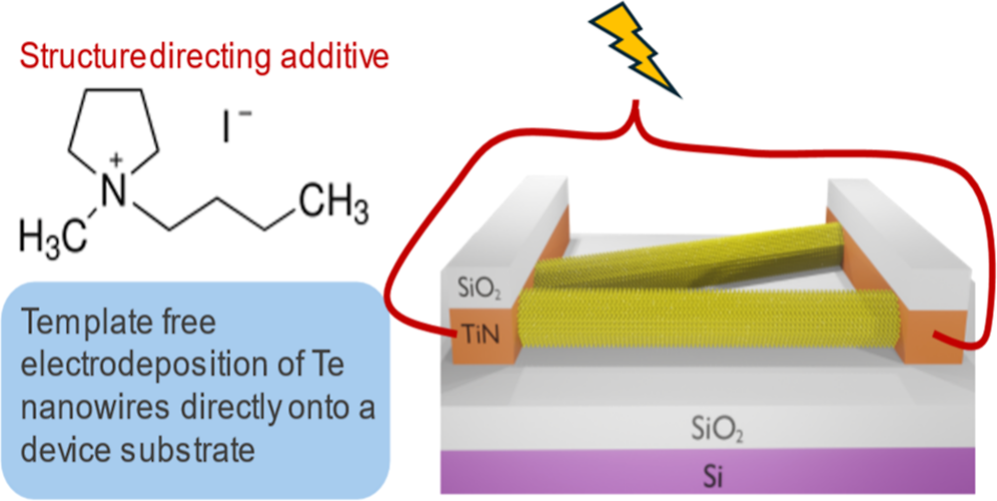

In this work, we present a method for direct, site-selective growth of tellurium nanowires by electrochemical deposition. The Te nanowires were grown laterally between two specially designed nanoband electrodes across a gap, and over a dielectric material, forming a lateral device structure directly. The resulting wires are crystalline and phase pure, as evidenced by Raman spectroscopy, EDS (energy dispersive X-ray spectroscopy), and ADF-STEM (annular dark field scanning transmission electron microscopy). The precise conditions for lateral growth of the nanowires were investigated and the fabrication of an electronic device from the as-deposited material, without the need for any transfer process or further contact fabrication, is demonstrated.

## 1 Introduction

Nanowires, typically defined as high aspect ratio structures with diameters between 1 and 200 nm, are unique physical systems that have been extensively studied because they exhibit properties such as one-dimensional quantum confinement of carriers and large surface-to-volume ratios.^1,2^ In addition, semiconductor industries have recently been exploring the possibility of using nanowires in gate-all-around transistor architectures for future electronic technologies.^3,4^ Despite these advances in nanowires, conventional techniques for their growth *via* methods such as molecular beam epitaxy (MBE) or metal–organic chemical vapor deposition (MOCVD) still lack control over the precise growth location. Template-based techniques to grow nanowires have emerged to tackle this issue but these typically require extra fabrication steps to pattern the template.^5,6^

On the other hand, elemental tellurium has attracted significant interest for applications in p-type field-effect transistors and mid-infrared photodetectors on both rigid and flexible substrates.^7,8^ It is an unusual element, as it is semiconducting and possesses a highly anisotropic crystal structure, a combination that gives rise to several interesting electronic properties.^9,10^ At room temperature and pressure, crystalline Te takes the form of covalently bonded helices with van der Waals forces between the chains of Te, a structure described as trigonal (t-Te).

In addition to the applications of bulk Te, the growth of tellurium nanowires is of high importance due to its potential in p-type gate-all-around architecture transistors and flexible mid-infrared photodetection.^7,10−14^ Work on the synthesis and properties of 2D and 1D forms of Te, consisting of a monolayer of Te and an individual helix, respectively, has also begun to emerge recently.^15−17^

Of the methods used to produce bulk Te and Te nanowires (Te NWs), electrochemical deposition (electrodeposition) has proven to be popular because of its simplicity, flexibility, and scalability. Electrodeposition is a “bottom up” approach, meaning that material growth starts from the electrode surface and proceeds outward, a feature that becomes particularly useful when producing materials with high aspect ratios. In previous works, we have used electrodeposition to demonstrate phase change memory, memristor and thermoelectric devices using bulk materials involving binary and ternary telluride alloys.^18−21^

Several reports of templated Te NW electrodeposition can be found in the literature, including deposition from aqueous electrolytes,^22−24^ and supercritical difluoromethane (CH_2_F_2_)^25^ into anodic alumina and track etch membrane templates. Achieving consistent nucleation and pore filling within the template can be a problem and so an approach for the fabrication of Te NWs that does not require a hard template is an attractive target. She *et al.* have reported template-free electrodeposition of Te NWs from TeO_2_ in 1 M KOH at 85 °C,^26^ and recently the template-free electrodeposition of Te NWs has been demonstrated from ionic liquids, including BMP-TFSI (1-butyl-1-methylpyrrolidinium bis(trifluoromethylsulfonyl)imide) containing SiCl_4_,^27^ EOPipTFSI/EOPipBr (1-ethyl-1-octylpiperidinium bis(trifluoromethylsulfonyl)imide/bromide mixture),^28^ and 12CE-Cl (choline chloride/ethylene glycol deep eutectic solvent) at temperatures between 40 and 100 °C.^29^ In all the above cases, further steps such as template dissolution and/or dispersion of the NWs in a solvent would be required for device fabrication, losing one of the inherent advantages of electrodeposition over chemical Te NW preparation.

In the present work, a method is presented for the direct growth of Te NWs across a SiO_2_ insulator between two nanoband electrodes, without the use of a template, by electrodeposition from dichloromethane (CH_2_Cl_2_) at room temperature. The nanowires are directly integrated with electrodes by electrodeposition so that no post-growth fabrication of contacts is required. This distinguishes our work from the previous reports described above and also other solution based methods of Te nanowire preparation,^13,16,30^ where typically the material is prepared separately and requires further processing such as transfer or lithography before device measurements are possible. The necessary conditions for successful NW electrodeposition are presented and the effect of the various experimental factors are discussed. The appearance and physical properties of the samples are studied using scanning electron microscopy (SEM), atomic force microscopy (AFM), annular dark field scanning transmission electron microscopy (ADF-STEM), energy dispersive X-ray spectroscopy (EDS) and Raman spectroscopy. Finally, the IV characterization of the resultant nanowire devices on the substrate is demonstrated.

## 2 Experimental

### 2.1 Chemicals

Dichloromethane, CH_2_Cl_2_ (95%, Sigma-Aldrich) was dried and degassed by refluxing with CaH_2_ under a dinitrogen atmosphere followed by distillation and were stored in an inert atmosphere of N_2_. The water content was measured with Karl–Fischer titration (KF 899 Coulometer, Metrohm, UK). There was less than 30 ppm of water in all solvents. Tetrabutylammonium chloride, [N^n^Bu_4_]Cl (Sigma-Aldrich, >99%) and 1-butyl-1-methylpyrrolidinium iodide (BMP-I) (Sigma-Aldrich, >98%) were dried by heating at 100 °C under vacuum for several hours. Tetrabutylammonium hexachlorotellurate (IV) was prepared using a method previously described in the literature.^31^ All solvents and reagents were stored in a dry N_2_ purged glovebox. Aqueous gold plating was performed using a commercial gold plating bath (“high speed gold”, Spa Plating, UK) without alteration.

### 2.2 Electrodes

The working electrodes were formed by fabricating TiN into micropatterned electrodes. The TiN electrodes were fabricated such that they are capped with a SiO_2_ layer to allow the material to grow laterally out from the edges of the electrodes, rather than over the top of them. To fabricate the lateral growth electrodes a photoresist was first spin-coated over a Si/SiO_2_ substrate and the desired pattern of the electrodes was transferred onto the photoresist by photolithography. 100 and 50 nm thick layers of TiN and SiO_2_ respectively were then sequentially sputtered and a lift-off process was used to form the final electrode structure.^32^ Each microelectrode was connected to a large global electrode at the top of the chip through which the electrochemical current is supplied. The TiN global electrode which provided electrical contact was capped with a 190 nm evaporated Au layer to reduce the contact resistance to the electrochemical setup. The chip has been described previously, with illustration, in ref^32^.

### 2.3 Electrochemistry

All glassware was cleaned by soaking in Decon 90 (Decon Laboratories Ltd., UK) for at least 24 h, followed by rinsing with ultrapure water, 0.055 μS cm^–1^ and then dried in an oven for a further 24 h. A Pt/Ir (90/10) disc was used as the counter electrode (CE) and the reference electrode (RE) was a homemade Ag/AgCl electrode stored in a 100 mM solution of [N^n^Bu_4_]Cl in CH_2_Cl_2_, separated from the electrolyte by a porous glass frit. Experiments were performed in a glovebox (Belle Technology, UK) under an inert atmosphere of N_2_ in the presence of <5 ppm O_2_ and H_2_O. A “front seal holder” arrangement was used to define the area of the working electrode, as described previously.^33^ Measurements were performed with a PGSTAT μIII (Metrohm Autolab, UK) potentiostat. Data were recorded with NOVA 1.11 (Metrohm Autolab, UK). The ambient temperature in the glovebox was monitored using a digital thermometer to an accuracy of ±0.05 °C (Hama, UK). Au pretreatment of the TiN electrode walls was performed in the same “front seal holder”, CE: Pt mesh, RE: saturated calomel electrode (SCE). A potential of −1.3 V *vs*. SCE was applied until a charge of 50 μC was passed.

### 2.4 Instrumentation

Scanning electron microscopy (SEM) was performed with ZEISS Sigma 500 VP FESEM. An Oxford instruments Ultim Max 170 mm^2^ was used for energy dispersive X-ray spectroscopy (EDS) and a WITec Raman RISE microscope for Raman spectroscopy. Raman spectroscopy was performed using a 532 nm laser at a power of 1 mW for 10 accumulations of 2 s. Polarized light microscopy was performed using a Nikon LV100ND (Nikon Metrology, UK) in bright field mode with episcopic illumination coupled with a polarizer and an analyzer. Images were captured using NIS elements software (Nikon Instruments, UK). Cross sectional TEM specimens were made by a focused ion beam (FIB) using a Tescan Amber FIB/SEM. Annular dark field images were taken from the Te nanowires using a JEOL ARM200f transmission electron microscope, operated at 200 kV. The current–voltage characteristics were measured at room-temperature using a semiconductor device analyzer (MSA-400).

## 3 Results and Discussion

**Figure 1:**
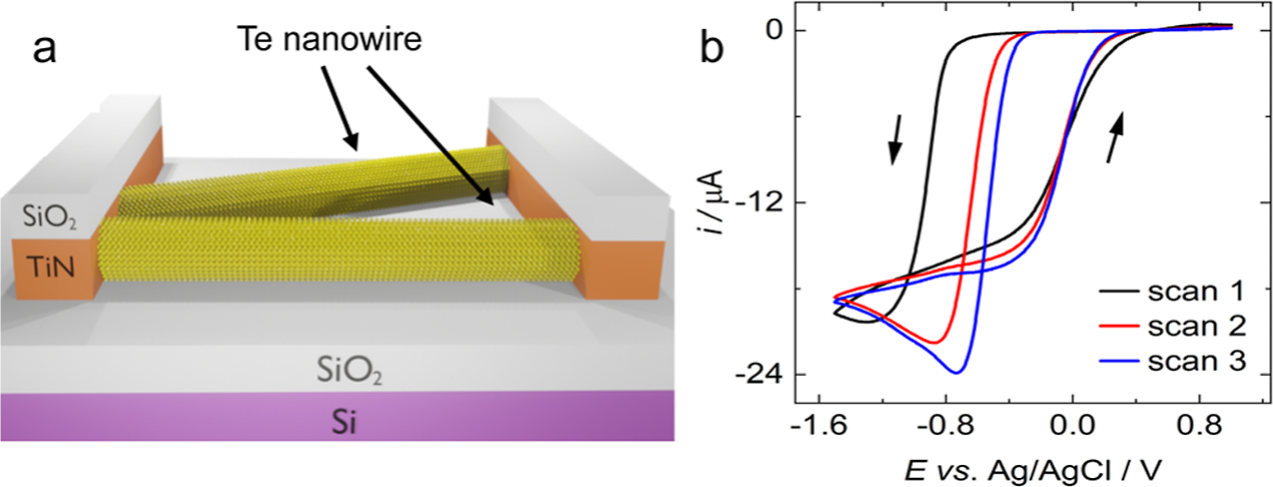
(a) Schematic showing the device structure for electrodeposited Te nanowires. (b) voltammograms of 0.75 mM [N^n^Bu_4_]_2_[TeCl_6_] and 100 mM BMP-I at a lateral growth electrode in CH_2_Cl_2_. Scan swept from 0.5 V *vs*. Ag/AgCl at 50 mV s^–1^ in the direction indicated by the arrows. CE: Pt mesh, RE: Ag/AgCl.

Figure 1a shows an illustration of the direct lateral growth of Te nanowires over a silica insulator by electrodeposition from the nanoband electrodes. This approach permits the electronic characterization of electrodeposited NWs directly on the substrate without need for any further modification or processing. The geometry of the substrate was exploited here to encourage Te to grow as nanowires from one electrode to the other. This also means that, in the context of an electronic device, the Te grows between two terminals. A similar electrode structure has previously been employed for lateral (in-plane) growth of a 2D material across an insulator, with minimal vertical (interplane) growth.^32^

(Figure 1b) shows a typical voltammogram at the TiN nanoband electrodes in an electrolyte containing 0.75 mM [N^n^Bu_4_]_2_[TeCl_6_] and 100 mM BMP-I in CH_2_Cl_2_. Scanning cathodically, a decrease in current can be observed at ca. −0.8 V *vs* Ag/AgCl associated with the electrodeposition of Te from [TeCl_6_]^2–^. On the reverse sweep, an apparent nucleation loop is present, but no stripping of the deposited Te appears to occur. With subsequent scans the onset of deposition takes place at higher potentials because of easier nucleation on an electrode where Te is already present. The electrochemistry of [N^n^Bu_4_]_2_[TeCl_6_] in CH_2_Cl_2_ at a planar TiN WE has been reported previously with a [N^n^Bu_4_]Cl supporting electrolyte.^31,34^ The voltammograms display similar features to those in (Figure 1b) with BMP-I, including comparable onset potentials, suggesting that the use of BMP-I and the nanoband electrode do not significantly affect the electrochemistry of Te^4+^.

**Figure 2:**
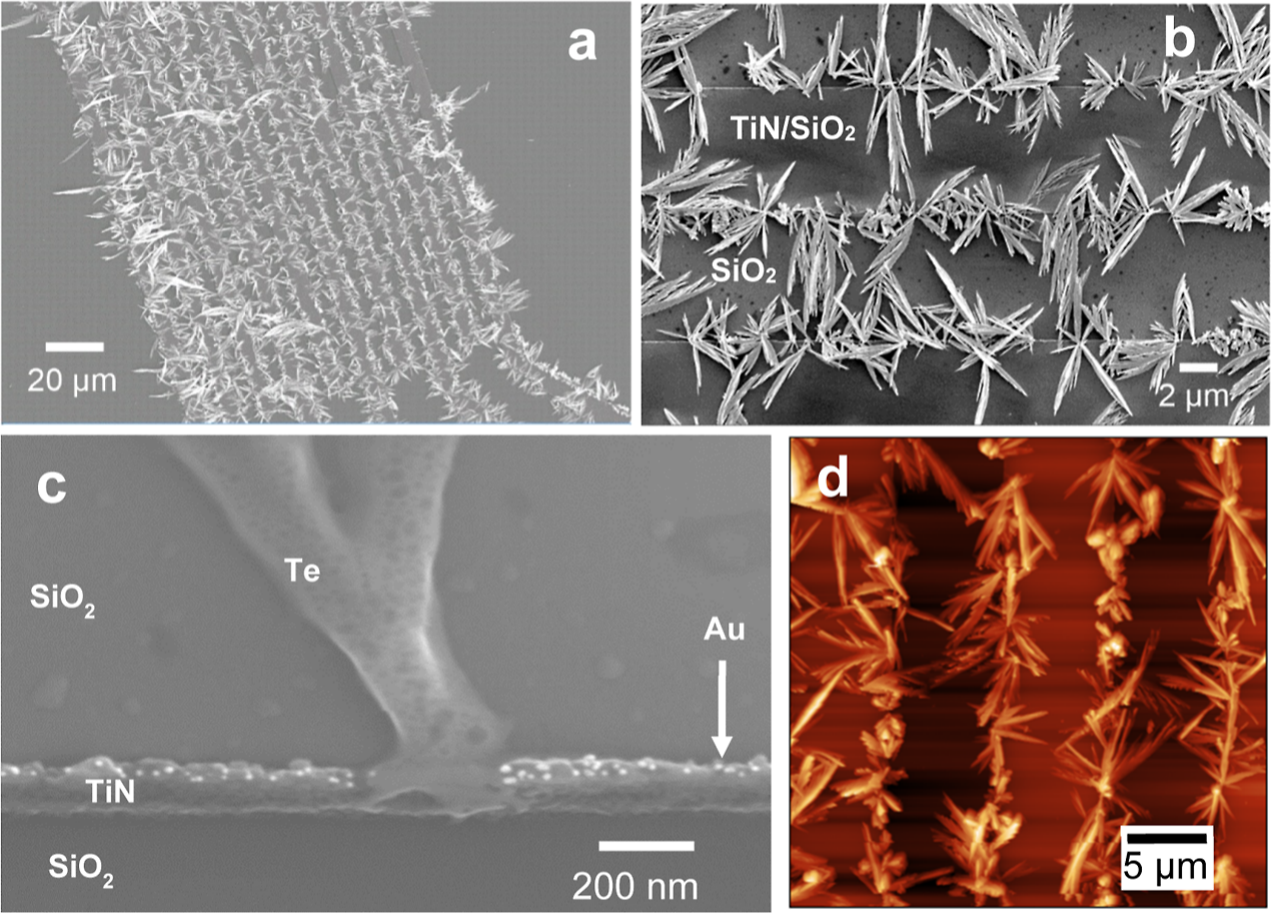
SEM and AFM images of electrodeposited Te NWs at various magnifications. (a) SEM image of the whole electrode array, (b) Inlens detector SEM image of a single channel within the array, (c) SEM image of an individual Te NW with pretreated Au nanoparticles visible, (d) AFM image of a single channel.

In order to achieve adequate Te NW growth, an initial pretreatment was required. First, a small amount of gold was electrodeposited on the electrode surface from a commercial aqueous Au plating bath. The purpose of this was to provide small Au sites where Te nucleation is more favorable than from TiN itself, and therefore encouraging the formation of nanowires rather than a film. It was also found to improve the homogeneity of nanowire growth across the electrode. As can be seen in (Figure 2c), the gold particles appear randomly distributed across the walls of the electrode and are *ca*. 5 nm in diameter. Second, a 2 s nucleation pulse at −1.75 V vs Ag/AgCl was used to initiate Te deposition. Due to the large cathodic overpotential of the pulse, the first step helps the Te to instantaneously nucleate on the substrate, and would also be expected to improve the uniformity and size distribution of the nanowires in the subsequent growth step, where no further nucleation takes place because of the lower applied overpotential.^35^

**Figure 3:**
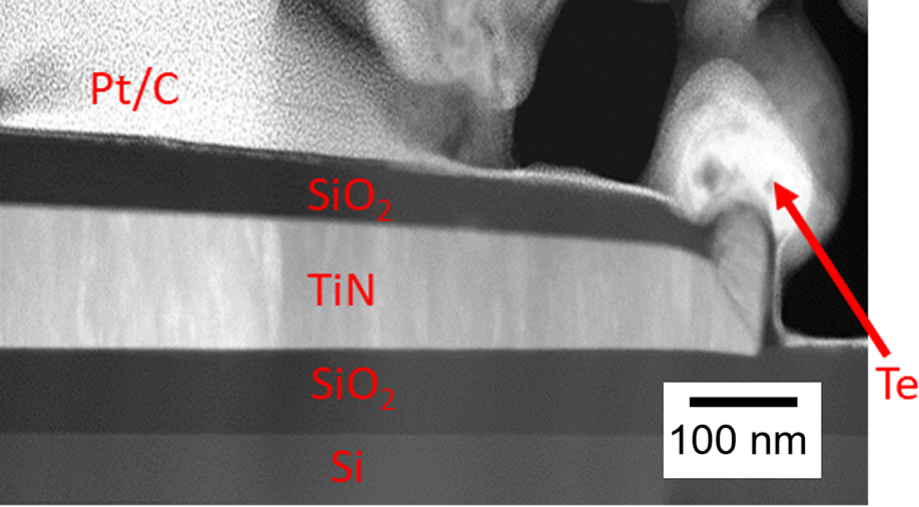
Cross-sectional ADF-STEM image of one electrode showing lateral growth of Te NWs. Pt and carbon were deposited on top of the device as a protection layer during FIB sectioning.

Figure 2 shows representative SEM and AFM images of Te NWs deposited onto the lateral growth substrate from an electrolyte containing 0.75 mM [N^n^Bu_4_]_2_[TeCl_6_] and 100 mM BMP-I for 300 s. A typical current transient for deposition under these conditions is shown in Figure S1. Te NWs can be observed to grow horizontally with a uniform distribution across the entire electrode. The wires appear to assemble into distinct units, clustering around a particular site and growing out and over the SiO_2_ surface in multiple directions. This is likely to be the result of the gold pretreatment and nucleation pulse where, as described above, the Au nanoparticles act as preferential sites for the growth of Te. The clustering effect causes some wires to grow in an undesirable direction over the top of the electrodes, but the majority cross the insulating gap and form the electrical contact necessary for electrical measurements. Lateral growth from the wall of the TiN substrate can also readily be observed with an ADF-STEM cross section, shown in Figure 3. The same image with EDS mapping showing the elemental composition of each location is given in Figure S2. Oxygen signals can be seen to coincide with Te in the maps, this is likely due to the presence of a passivating surface oxide film, this could be studied in further detail with selected area electron diffraction (SAED) and X-ray photoelectron spectroscopy (XPS). The formation of bulk TeO_x_ can excluded by the use of a nonaqueous solvent with negligible dissolved oxygen.

There is some variation in the length and diameter of the Te NWs, larger deposits appear needle-like in places with some branching and “feathering” (*e.g.*, Figure 2c), can also be observed. The NWs are typically in the range of 1–7 μm in length and *ca*. 200 nm in diameter.

Attempts were made to image Te NWs that had grown directly from the Au nanoparticles to verify our hypothesis that the gold nanoparticles provided nucleation sites for Te NW growth. However, this was challenging, not only due to the small size of the gold nanoparticles, but also because the Te may grow over it and so block the Au from view, as indicated in Figure 2c. Nevertheless, it is occasionally possible to observe direct growth, such as in Figure S3, which shows SEM imaging and EDS maps of Te grown from a gold particle on the lateral growth electrode. It is possible for Te to nucleate and grow from sites other than the pretreated Au, however the SEM images in Figures 2 and S3 indicate that the majority grow from individual Au particles.

In summary, our preliminary studies show that the conditions for good growth of Te NWs across the insulating surface are as follows: a lateral growth substrate, use of BMP-I as the supporting electrolyte, pretreatment to form Au nanoparticles and application of a nucleation pulse prior to bulk deposition. Results for depositions in which one of these steps was omitted are shown in Figure S4, in each case nucleation is poor and minimal lateral growth of Te NWs is observed.

It is also important to emphasize that the effect of the Au pretreatment does not imply that the same results would be observed using an Au electrode in place of TiN. The Au pretreatment provides small islands of a more active metal relative to the TiN surrounding it and this, combined with the nucleation pulse, means that the Te will preferentially nucleate at those sites and then grow laterally. Nanowires are still formed without the pretreatment (*cf*. Figure S4), but this step asserts some control over the process and encourages growth in a more ordered fashion, laterally across the insulator, rather than randomly.

**Figure 4:**
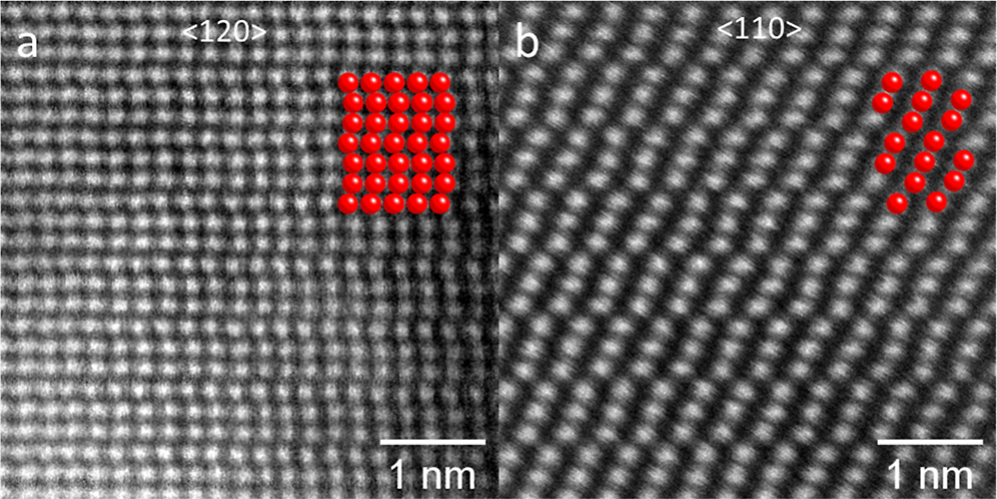
High resolution ADF-STEM images of electrodeposited Te NWs deposited with the optimized conditions, taken along the (a) < 120> and (b) < 110> directions, respectively. Structural models of bulk Te crystals projected along the same directions are overlaid (red), which confirm that the Te NWs adopt the structure of the bulk Te crystal.

Having established suitable conditions for Te NW growth, the wires were subjected to various characterization methods to further understand their physical properties. Figure 4 shows high resolution ADF-STEM images taken from the Te NWs along either the <120> or the <110> direction. Structural models of bulk Te crystals projected along the same directions are also shown along with the images, which confirm at the atomic scale that the Te NWs have adopted the crystal structure of bulk Te. Typically X-ray diffraction can be used to assess the crystallinity of the material, however this was not suitable in the present work because of the limited sample size, hence ADF-STEM was used.

**Figure 5:**
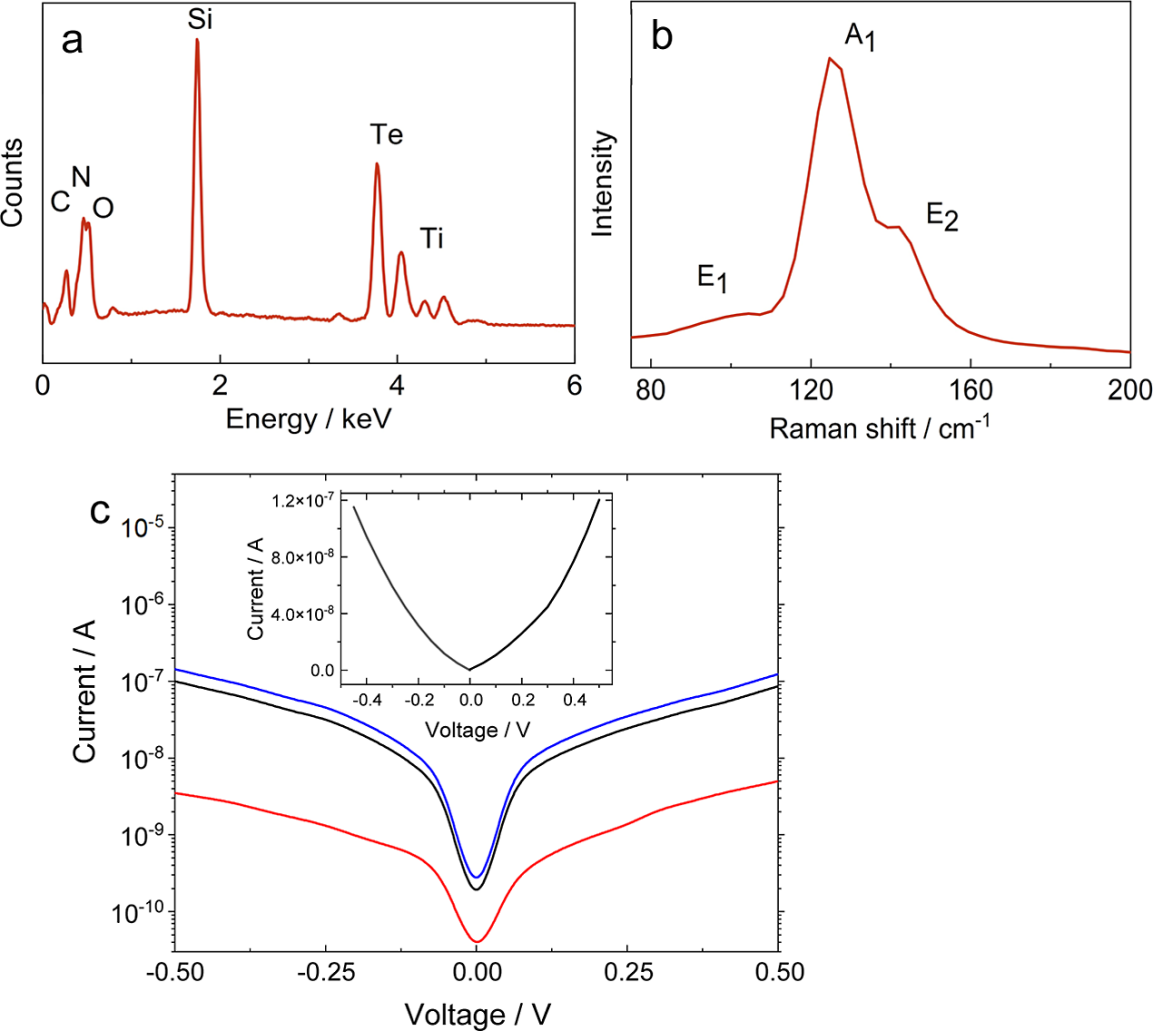
Physical and electronic characterization of Te NWs deposited under the optimized conditions showing (a) representative EDS spectrum, (b) Raman spectrum collected with a 532 nm laser, and (c) current–voltage sweep, where each color represents an individual device, inset is the linear scale showing one device.

Figure 5a shows a representative EDS spectrum, confirming the presence of elemental Te, along with a representative Raman spectrum in 5b. No EDS signals corresponding to Cl or I are observed. O, Si and Ti are associated with the substrate and the C signal can be attributed to adventitious carbon. Crystalline bulk Te shows characteristic Raman peaks at 92, 120 and 141 cm^–1^ corresponding to the *E*_1_, *A*_1_ and *E*_2_ Raman modes respectively.^36^ These are each associated with bond bending, chain expansion and bond stretching, respectively. Here, a peak is observed at 126 cm^–1^, which can be attributed to the *A*_1_ mode. A shoulder is then observed at 142 cm^–1^, associated with the *E*_2_ mode. A weak, broad feature also occurs at 104 cm^–1^, which corresponds to the *E*_1_ mode. A blueshift in the Te Raman peaks (particularly in the *A*_1_ mode) relative to bulk Te has been reported previously for nanostructured Te, and it has been suggested that this occurs because of a weakening of the van der Waals interactions between Te chains, and strengthened intrachain covalent interactions.^13,30^ The *E*_2_ mode tends to shift to higher wavenumbers by a lesser extent than the *A*_1_, possibly explaining why it morphs into a shoulder of the *A*_1_ peak in the spectrum in Figure 5. It also indicates that there is indeed a degree of nanostructuring in the Te NWs prepared in this work. Background Raman spectra of TiN and SiO_2_ (Figure S5) confirming that they are Raman inactive within the range measured.

The response of the Te NWs to polarized light can give an indication of the presence of chirality, as shown in Figure S6 with images of unpolarized and cross-polarized light. By setting the polarizer and analyzer perpendicular to each other, the light is polarized both vertically and horizontally (cross-polarized), and no light passes through. However, if an anisotropic *i.e.* chiral material is illuminated, the polarized light vector undergoes rotation within the sample, allowing a proportion of its wave vector to pass through the analyzer, resulting in observable features and a qualitative indication of the presence or absence of an anisotropic material. As can be seen, the Te NWs appear brighter with cross-polarized light, which could suggest the existence of chirality, as would be expected from the crystal structure of Te. Polarized light microscopy is typically employed to identify birefringence in crystals, and has been previously used to detect chirality in Te nanostructures,^37^ where samples which were chiral in polarization dependent second-harmonic generation spectroscopy were also shown to be birefringent with polarized light microscopy.

The results of current–voltage IV sweep on the as-deposited Te NWs are displayed in Figure 5c for three different devices. The lateral growth electrodes, positioned at the bottom of the chips, are initially connected to a global contact where the potential for electrodeposition is applied. Afterward, the chips are cleaved into two-halves along the cleave zone before conducting electrical measurements. This step is essential to isolate the electrodes. The chip layout and methodology used in this study closely follow the designs established in our previous work.^32^ The data shows that the Te NWs have grown across the SiO_2_ insulating gap to form contacts between the two TiN nanoband electrodes, and therefore demonstrates the possibility of fabricating an electronic device without any further modification of the substrate. The current in different devices can vary, depending on the number and size of Te wires bridging the electrodes. The room temperature average resistivity is measured to be 1.17–5.85 × 10^3^ Ω cm. The typical device dimensions used are as follows: channel length (L): 8 μm, channel width (W): 200 μm. Only a few studies have reported resistivity values for Te nanowires, ranging from around 10 to 50 Ω cm.^38,39^ These reported values are indeed lower than those we are observing. This discrepancy can be attributed to several factors related to composition, crystallinity, our device geometry and the specifics of the electrodeposition process. Further analysis and experimentation would be necessary to fully understand the implications of these parameters on the device functionality, but this highlights the ability of the nanoband electrodes to measure electrical responses directly from the electrodeposited Te NWs, without having to perform post deposition patterning or fabrication.

## 4 Conclusions

This work has presented a novel method for the fabrication of Te NWs from Au nanoparticles and across an insulator by electrodeposition onto TiN nanoband electrodes, without the need to use a template. The anisotropic nature of Te is exploited, and good nanowire growth can be achieved using a set of conditions designed to promote nucleation and lateral electrodeposition of Te. The protocol includes: a specifically designed lateral growth substrate, use of the supporting electrolyte BMP-I, a pretreatment of the TiN electrode surface with particles of Au by electrodeposition, and an initial nucleation pulse. The nature of the deposited Te NWs and the effect of the various experimental conditions were probed with SEM, while the presence of crystalline, elemental Te was confirmed with ADF-STEM, EDS and Raman spectroscopy. An added advantage of the lateral growth substrate is that electronic measurements could be made directly on the substrate, permitting study of the Te NWs as they were deposited, without the need for processing post hoc. The formation of an electronic device was then demonstrated through current–voltage sweeps.

The key factor in determining the presence of lateral growth, and therefore the formation of Te NWs, seems to be the presence of BMP-I rather than [N^n^Bu_4_]Cl in the electrolyte. It is possible that BMP-I is acting as a structure directing additive, controlling the morphology of the Te and promoting growth in a specific direction so causing the formation of nanowires. It is not presently clear why this is, but since the applied potential is likely to be more negative than the potential of zero charge (pzc), the double layer will be populated with the BMP cation. It is possible that this species adsorbs onto the electrode surface, or onto the depositing Te, and influences the direction of growth.

The development of this method provides a number of interesting opportunities for further work. In nanoparticle synthesis, a capping agent, such as polyvinylpyrrolidone (PVP), is often used to control the size and shape of the resulting particle. This may also be beneficial here, to increase the uniformity of the Te NW growth, as well as reducing the “feathering” that is sometimes observed. Furthermore, one of the most interesting features of Te NWs is their chirality, and the ability to have enantiomeric control during the electrodeposition process would be highly advantageous. This might be possible by using a chiral Te precursor or chiral additives. Finally, as described above, nanostructured Te is of interest as a transistor material and so future work will focus on fabricating transistors with the electrodeposited Te NWs.

## Supporting Information Available

Representative current transient for electrodeposition of Te nanowires including nucleation pulse and bulk deposition; STEM cross-section of electrodeposited Te nanowires with added EDS mapping; SEM image and EDS mapping indicating Te nanowires growing directly from an Au particle; SEM images showing the effect of removing each step in the deposition process which is required for good Te nanowire growth; Raman spectra of electrodeposited Te nanowires over a wider wavenumber range and background spectra of SiO_2_ and TiN (PDF)
