# Supplementary material for: Toward a Procedure for the Template Free Growth of Te Nanowires Across an Insulator by Electrodeposition
Source: J Phys Chem C Nanomater Interfaces. 2024 Oct 22;128(43):18634–40. doi: 10.1021/acs.jpcc.4c05915 (PMC11533211; doi:10.1021/acs.jpcc.4c05915)
Supplement: Supplementary file 1 — jp4c05915_si_001.pdf [file jp4c05915_si_001.pdf]

Towards a Procedure for the Template Free Growth of Te Nanowires Across an Insulator by Electrodeposition

Alexander W. Black,[a] Nema M. Abdelazim,[b]* Yasir J. Noori, [b] Yisong Han,[c] Nikolay Zhelev,[a] Shibin Thomas,[a] Wenjian Zhang,[a] Gillian Reid,[a] Richard Beanland,[c] C. H. Kees De Groot,[b] Philip N. Bartlett[a]*

[a]School of Chemistry, University of Southampton, Southampton, SO17 1BJ, UK, [b]School of Electronics and Computer Science, University of Southampton, SO17 1BJ, UK, [c]Department of Physics, University of Warwick, Coventry, CV4 7AL, UK

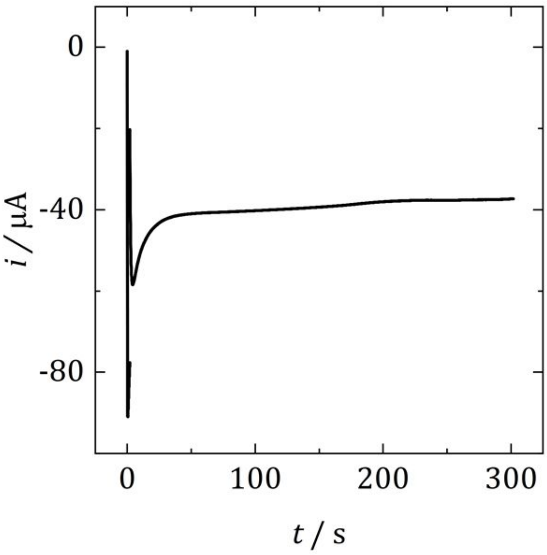

Figure S1: representative current transient for the electrodeposition of Te NWs obtained using an electrolyte composed of 0.75 mM $[N^nBu_4]_2[TeCl_6]$ and 100 mM BMP-I in CH_2Cl_2. The potential was stepped to -1.75 V for 2 s then to -0.4 V for 300 s. WE: TiN lateral growth substrate, CE: Pt disc, RE: Ag/AgCl.

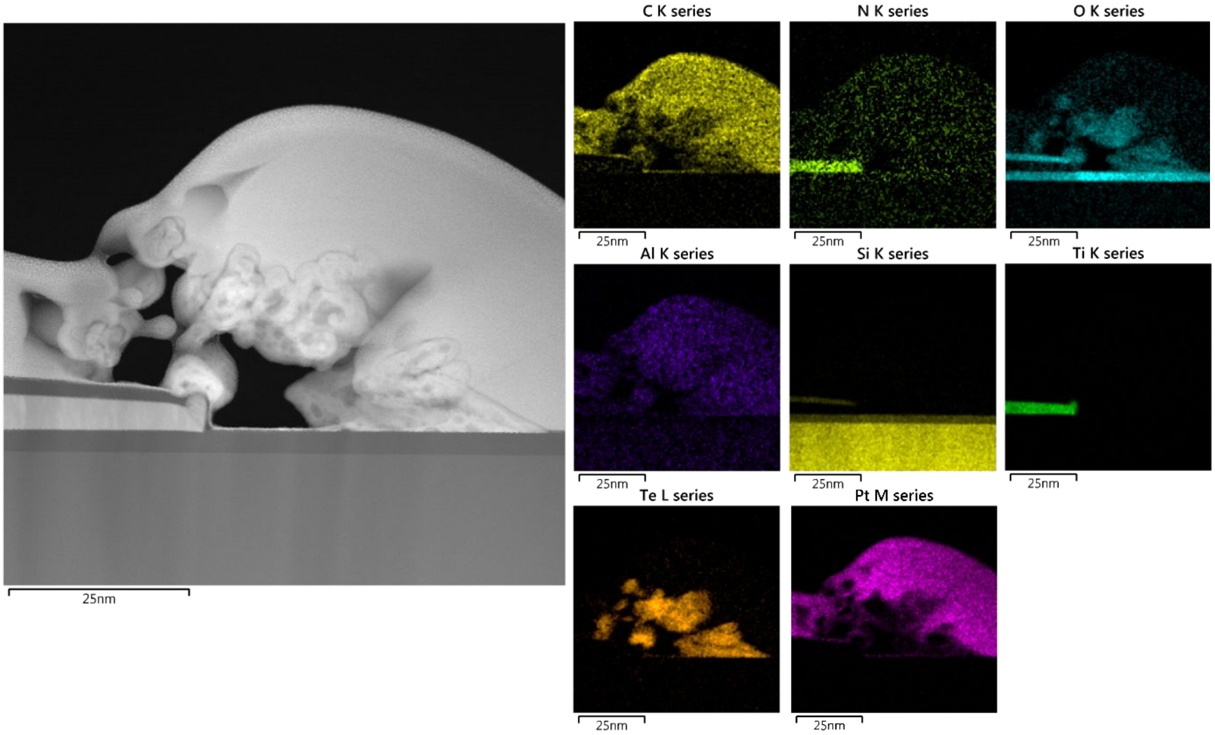

Figure S2: STEM cross-section of electrodeposited Te NWs, along with EDX mapping showing the elemental composition at each location.

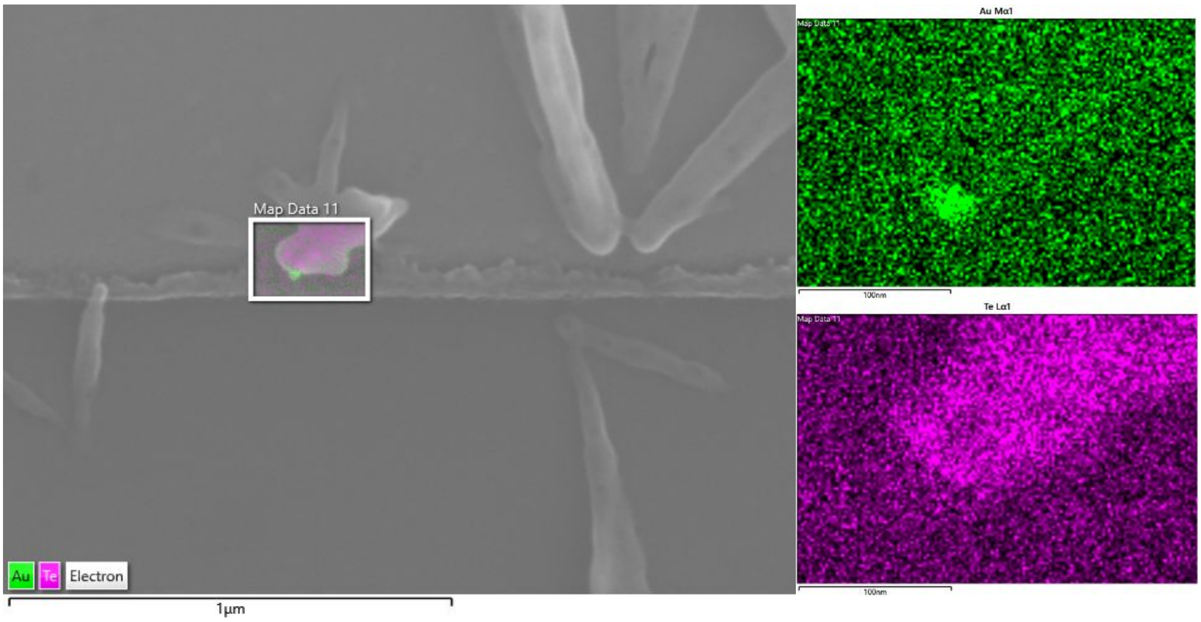

Figure: S3: EDS map showing Te growth directly from pre-treated Au.

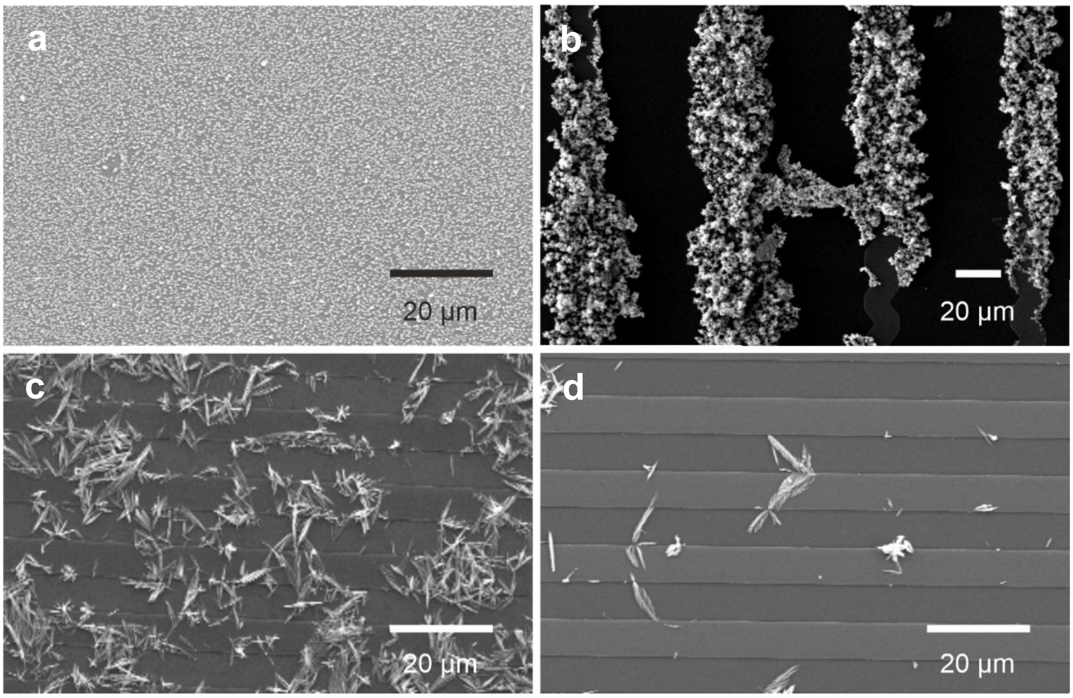

Figure S4: SEM images showing the effect of removing various experimental steps on the electrodeposition of Te NWs. a) without a lateral growth substrate, deposited onto a disc electrode, b) without BMP-I, deposited with 100 mM [N^nBu_4]Cl as the supporting electrolyte, c) without Au pre-treatment, d) without a nucleation pulse.

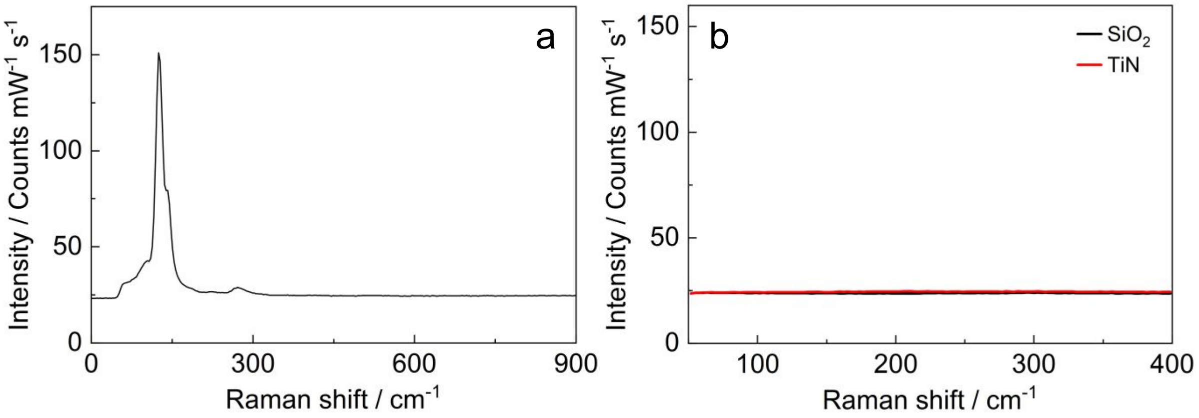

Figure S5: representative Raman spectra of a) wider range of electrodeposited Te, b) regions of SiO_2 and TiN on lateral growth substrates. collected with a 532 nm laser.

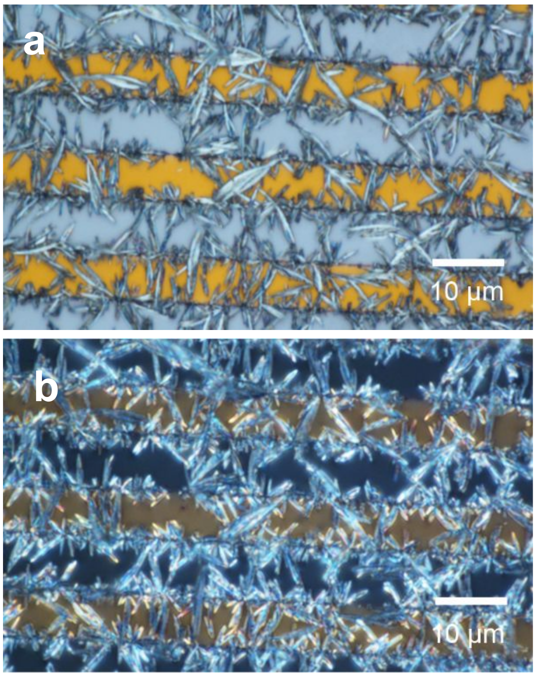

Figure S6: The effect of polarized light on optical micrographs of electrodeposited Te NWs with a) unpolarized and b) cross-polarized light.
